# Modeling Current Sources for Neural Stimulation in COMSOL

**DOI:** 10.3389/fncom.2018.00040

**Published:** 2018-06-08

**Authors:** Nicole A. Pelot, Brandon J. Thio, Warren M. Grill

**Affiliations:** ^1^Department of Biomedical Engineering, Duke University Durham, NC, United States; ^2^Department of Electrical and Computer Engineering, Duke University Durham, NC, United States; ^3^Department of Neurobiology, Duke University Durham, NC, United States; ^4^Department of Neurosurgery, Duke University School of Medicine Durham, NC, United States

**Keywords:** computational modeling, neural engineering, finite element method, boundary conditions, neuromodulation

## Abstract

**Background:** Computational modeling provides an important toolset for designing and analyzing neural stimulation devices to treat neurological disorders and diseases. Modeling enables efficient exploration of large parameter spaces, where preclinical and clinical studies would be infeasible. Current commercial finite element method software packages enable straightforward calculation of the potential distributions, but it is not always clear how to implement boundary conditions to appropriately represent metal stimulating electrodes. By quantifying the effects of different electrode representations on activation thresholds for model axons, we provide recommendations for accurate and efficient modeling of neural stimulating electrodes.

**Methods:** We quantified the effects of different representations of current sources for neural stimulation in COMSOL Multiphysics for monopolar, bipolar, and multipolar electrode designs.

**Results:** We recommend modeling each electrode contact as a thin platinum domain, modeling the electrode substrate with the conductivity of silicone, and either using a point current source in the center of each electrode contact or using a boundary current source. Alternatively, to avoid possible numerical instabilities associated with a large range of conductivity values (i.e., platinum and silicone) and to eliminate the small mesh elements required for thin electrode contacts, the electrode substrate can be assigned the conductivity of platinum by using insulating boundaries between the substrate and surrounding medium, and within the substrate to isolate the contacts from each other. When modeling more than one contact, we recommend using superposition by solving the model once for each contact, leaving inactive contacts floating, and superposing the resulting potentials. We computed comparable errors in activation thresholds across the different implementations in a simplified model (electrode in a homogeneous, isotropic medium), and in realistic models of rat spinal cord stimulation (SCS) and human deep brain stimulation, indicating that the recommended approaches are applicable to different stimulation targets.

## Introduction

Computational modeling provides an important toolset for designing and analyzing neural stimulation devices to treat neurological disorders and diseases. Modeling enables efficient exploration of large parameter spaces, where preclinical and clinical studies would be infeasible. The typical workflow for computational modeling of a neural stimulation device involves a three-dimensional finite element model (FEM) of the electrode and nearby tissues to calculate the distribution of electric potentials in the tissues (e.g., to study deep brain stimulation Howell and McIntyre, 2017, SCS Lempka et al., 2015, and peripheral nerve stimulation Pelot et al., 2017). Potential distributions are then applied as inputs to non-linear cable models of the neurons to calculate the resulting changes in transmembrane potential or other outcome measures (e.g., threshold for activation).

Current commercial finite element method software packages (e.g., COMSOL Multiphysics, ANSYS) enable straightforward calculation of the potential distributions. However, it is not always clear how to implement boundary conditions to represent appropriately metal stimulating electrodes. Published models of neural stimulation devices used different boundary conditions to model current source electrodes and many lack sufficient detail regarding the implementation (e.g., Butson and McIntyre, 2005; Helmers et al., 2012; Howell et al., 2014; Lempka et al., 2015). Therefore, we quantified the effects of different representations of current sources for neural stimulation in COMSOL Multiphysics for monopolar, bipolar, and multipolar electrode designs. By quantifying the effects of different electrode representations on activation thresholds for model axons, we provide recommendations for accurate and efficient modeling of neural stimulating electrodes.

## Methods

### Model description

**Figure 1:**
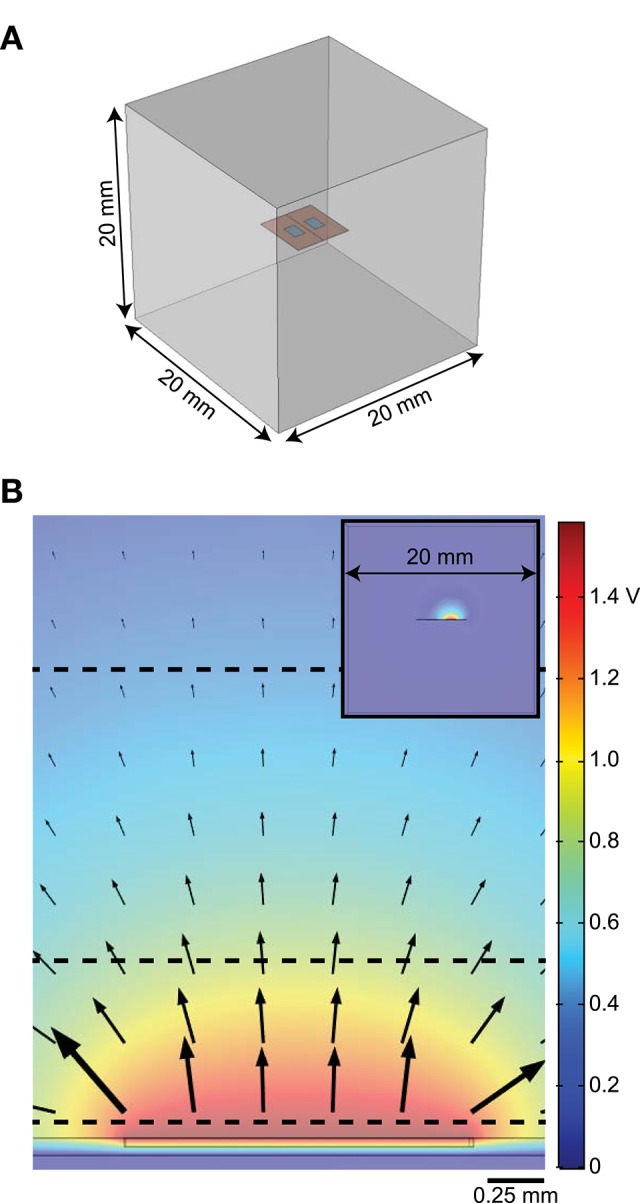
Diagrams of finite element model (FEM). **(A)** 3D FEM showing electrode substrate (orange) with two contacts (blue) in a homogeneous medium (gray). The line along the center of the orange substrate, between the electrodes, illustrates the location of the workplane used as an insulating boundary when needed (i.e., when the electrode substrate is assigned the conductivity of platinum). **(B)** Distribution of potentials in the tissue with one active contact (floating potential boundary condition) and the outer boundary of the FEM set to ground. The arrows show the direction of current out of the stimulating electrode and the black dashed horizontal lines indicate where model axons were positioned. The inset shows the cross-section of the whole model.

We built a simplified three-dimensional FEM of an electrode in tissue using COMSOL Multiphysics v5.3 (COMSOL Inc., Burlington, MA) and calculated potentials assuming quasi-static conditions (Bossetti et al., 2008). We coupled the FEM-calculated voltages to non-linear cable models of myelinated axons to calculate activation thresholds in response to different representations of the modeled current source(s). We tested monopolar (1 active contact), bipolar (2 active contacts), and multipolar (>2 active contacts) configurations. For the monopolar and bipolar configurations, the model geometry consisted of a planar bipolar electrode in a box (Figure 1). The electrode contacts were 1.5 mm long × 1 mm wide × 0.025 mm deep and spaced 2 mm apart center-to-center (1 mm edge-to-edge). We embedded the contacts in a 5 mm × 5 mm × 0.05 mm silicone sheet (“electrode substrate”), consistent with the dimensions of electrodes used for rat SCS (Crosby et al., 2017). We created a workplane within the electrode substrate, halfway between the contacts and orthogonal to their surface (Figure 1A); as described below, we only used the workplane as an insulating boundary when modeling the entire electrode as a block of platinum, but otherwise assigned a condition of continuity. We placed the electrode in the center of a conductive box (20 mm × 20 mm × 20 mm). We set the conductivity of the medium to that of muscle (0.2 S/m) (Geddes and Baker, 1967). We grounded the outer boundaries of the box (V = 0). To assess computational efficiency, we recorded the time required to solve the FEM on an Intel Core i7-5700HQ CPU at 2.70 GHz with 16 GB of RAM, running Windows 10 Home Edition.

We exported the electric potentials from the FEM at three electrode-axon distances (0.05, 0.75, and 2 mm; dashed lines in Figure 1B) with the model axons centered over the electrode contacts. These distances span the range of relevant distances in rats and humans; for example, the diameter of the cervical vagus nerve is ~3 mm in humans (Verlinden et al., 2016) and ~0.25 mm in rats (Licursi de Alcântara et al., 2008), while the thickness of the dorsal cerebrospinal fluid in the lower thoracic region (i.e., distance from an epidural spinal cord stimulator to the superficial dorsal column fibers) is ~5 mm in humans (Holsheimer et al., 1994) and ~0.3 mm in rats (in-house MR image). We applied the potentials to 20 mm-long models of mammalian axons using the McIntyre-Richardson-Grill (MRG) model (2, 5.7, and 10 μm diameter axons) (McIntyre et al., 2002, 2004) implemented in NEURON v7.4 (Carnevale and Hines, 2006). The nodes of Ranvier in the MRG model contain a non-specific leak current and three nonlinear voltage-dependent ion channels (fast sodium, persistent sodium, and slow potassium), as well as finite linear conductance and capacitance at the paranodal and internodal segments. The geometrical and electrical parameters were all based on measurements of mammalian nerve fibers. We used a binary search algorithm to find activation thresholds (with 1 μA resolution) for a symmetric biphasic cathodic-first pulse with 200 μs per phase.

We meshed the FEM using tetrahedral elements with quadratic geometry and solution shape functions. After doubling the volume of the outer domain or increasing the number of domain mesh elements from 330,020 to 837,844, the peak potential of each axon location and diameter changed by <2% and the activation thresholds changed by <1%. Therefore, we concluded that our mesh density and model volume were sufficient. We meshed the model once (330,020 elements) and solved it multiple times with different boundary conditions to avoid changes in thresholds due to changes in the mesh. We only re-meshed the model when we changed the geometry [i.e., changing from flush to raised contacts (333,576 elements) or modeling the contacts without a thin domain (322,581 elements].

### Monopolar

We varied four parameters to determine their effects on axon activation thresholds and on FEM solution convergence time for nine combinations of model axon diameter and electrode-axon distance:

1. **Contact geometry:**
  - Surface of the electrode contact flush with the surface of the electrode substrate
  - Electrode contact half embedded and half raised with respect to the surface of the electrode substrate
2. **Contact model:**
  - Thin platinum domain used to represent each contact
  - Thin domain used to represent each contact assigned the same conductivity as the electrode substrate
  - No thin domain: each contact represented as a boundary condition without a physical representation of the metal contact volume
3. **Source:**
  - PCS: point current source (specify current)
  - Active boundaries
    - EP: electric potential source (specify voltage; Dirichlet boundary condition)
    - BCS: boundary current source (specify current density; Neumann boundary condition)
    - FP: floating potential source (specify current)
4. **Electrode substrate:**
  - σ_Sil_ (Sil: silicone)
  - Electrically insulated: perfectly insulated boundaries for all substrate surfaces contacting the surrounding tissue medium. Conductivity within the substrate domain:
    - σ_Sil_
    - σ_medium_
    - σ_Pt_ with insulating boundary between the contacts (aforementioned workplane)

**Figure 2:**
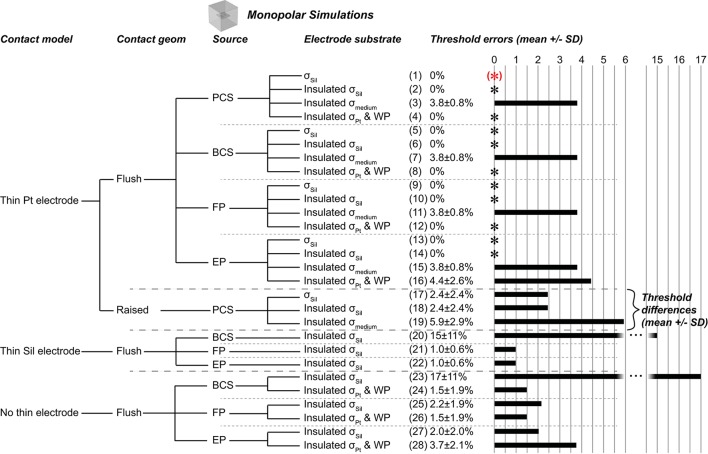
Monopolar test cases. (*)Gold standard used to compute errors in thresholds (mean ± *SD*) across nine combinations of axon location and diameter. The black asterisks denote cases with zero error as compared to the gold standard. Source models: point current source (PCS), boundary current source (BCS), floating potential (FP), electric potential (EP). Materials: silicone (Sil), platinum (Pt), medium (muscle). WP: Insulated workplane separating contacts electrically within the electrode substrate. All thresholds shown in Supplementary Table 1.

We evaluated select combinations of these four parameters (Figure 2). The conductivity values (σ) were σ_Sil_ = 10^−12^ S/m (Davis, 1959), σ_medium_ = 0.2 S/m (Geddes and Baker, 1967), and σ_Pt_ = 9.43^*^10^6^ S/m (Serway, 2006).

For all monopolar simulations, only one of the two electrode contacts was active and delivered 1 mA of current. The inactive contact had no assigned source and was simply assigned the same geometry and material properties as the active contact. We evaluated four different source models. First, we placed a 1 mA point current source in the center of the platinum domain. The other three sources were active boundary conditions assigned to the single surface between the electrode and the medium for the flush contact geometry; approaches for the raised contact geometry are discussed below. The floating potential implementation simply required setting the output current to 1 mA. Using the electric potential source involved two steps. First, we set the voltage of the electrode contact to 1 V. We then integrated the normal current density (Equation 1) over the grounding box, i.e., the outer boundaries of the model:

(1) $$\begin{array}{r} \text{ec}.\text{normJ}=\text{ec}.\text{J}{\text{x}}^{*}\text{nx}+\text{ec}.\text{J}{\text{y}}^{*}\text{ny}+\text{ec}.\text{J}{\text{z}}^{*}\text{nz} \end{array}$$

Because the model was purely conductive, the voltage of the electrode contact was linearly related to the delivered current. We then solved the model a second time with the voltage of the electrode contact set to the reciprocal of the integrated current density in milliamps, resulting in a source that output 1 mA. Alternatively, we could scale the potentials resulting from a 1 V source by the applied current rather than re-solving the model. Lastly, when using the boundary current source, we set the current density of the electrode to the reciprocal of the active contact's exposed surface area. As verification for all four source models, we integrated the current density over the model's outer boundaries, to ensure that 1 mA of current was delivered by the electrode contact.

When using the raised contact geometry, we evaluated each of the four source models with minor modifications. We placed the point current source in the center of the contact volume. For the floating potential, we assigned the boundary condition to all five of the surfaces of the contact exposed to the medium and set it to 1 mA. Similarly, for the electric potential approach, we assigned 1 V to all five of the exposed surfaces of the contact. Lastly, for the boundary current source approach, we assigned the boundary current source the value of 1 mA divided by the total exposed surface area of the contact. For each approach, we verified that the total integrated current density over the outer boundaries was 1 mA.

### Bipolar

**Figure 3:**
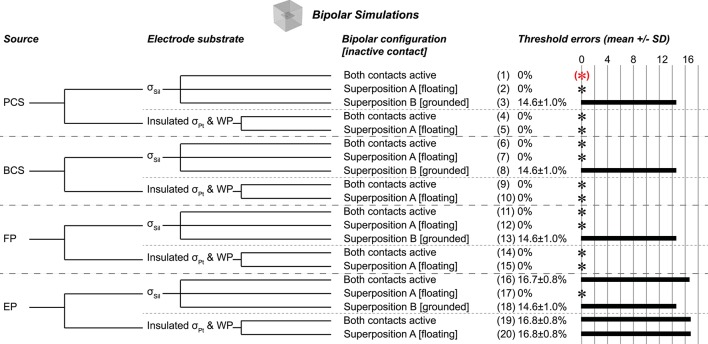
Bipolar test cases, all using flush contact geometry with thin platinum electrode contacts. (*)Gold standard used to compute errors in thresholds (mean ± *SD*) across nine combinations of axon location and diameter. The black asterisks denote cases with zero error as compared to the gold standard. Source models: point current source (PCS), boundary current source (BCS), floating potential (FP), electric potential (EP). Materials: silicone (Sil), platinum (Pt). WP: Insulated workplane separating contacts electrically within the electrode substrate. Superposition A: Inactive contact is floating as in the monopolar simulations. Superposition B: Inactive contact is grounded. All thresholds shown in Supplementary Table 2.

We quantified the effects of different representations of a bipolar current source by implementing key test cases based on the results from the monopolar scenarios (Figure 3). We used the same geometry and mesh as for the monopolar simulations. We obtained excitation thresholds for model axons using both contacts active simultaneously (+1 mA for one contact and −1 mA for the other contact) or using one contact active at a time and then superposing the resulting potential distributions. More specifically, when testing superposition, one electrode contact delivered +1 mA while the other electrode contact was inactive. We then ran the simulation a second time with the other electrode contact delivering −1 mA while the first electrode was inactive. We then summed the resulting potential distributions. Two conditions for the inactive contact were compared, either floating (with a condition of continuity, as used in the monopolar simulations) (Superposition A) or grounded (Superposition B).

We again evaluated all four source models as described for the monopolar simulations (PCS, BCS, FP, EP), except for the case of the electric potential source when both contacts were active simultaneously. In this case, we determined the required boundary voltage to deliver the target current (±1 mA) for each contact separately, then applied the voltages simultaneously.

### Multipolar

**Figure 4:**
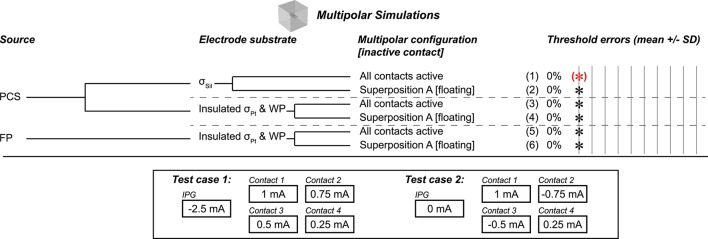
Multipolar test cases, all using flush contact geometry with thin platinum electrode contacts. (*)Gold standard used to compute errors in thresholds (mean ± *SD*) across nine combinations of axon location and diameter. All cases resulted in zero error as compared to the gold standard, as denoted by the black asterisks. Source models: point current source (PCS), floating potential (FP). Materials: silicone (Sil), platinum (Pt). WP: Insulated workplane separating contacts electrically within the electrode substrate. Superposition A: Inactive contact is floating as in the monopolar simulations. The bottom panel shows the current configurations that we evaluated; the axons were centered over the four contacts, coursing horizontally. All thresholds shown in Supplementary Table 3.

We extended the model from a two-contact configuration to a four-contact configuration with the contacts placed in a 2 × 2 grid. We spaced adjacent electrode contacts 2 mm apart center-to-center, and we extended the electrode substrate to 10 mm × 5 mm × 0.05 mm. We maintained the dimensions of the contacts and of the surrounding box, resulting in 537,174 tetrahedral elements. We ran key test cases based on the results from the bipolar simulations with and without current sinking to the outer boundaries of the model (intended to represent current return to the enclosure of an implanted pulse generator); we tested configurations with net zero current and with net non-zero current across the contacts, with current returned to the boundary in the latter case (Figure 4). We compared thresholds with all contacts active simultaneously to thresholds using superposition of the potential distributions with the inactive contacts floating (condition of continuity on their surfaces).

### Model of spinal cord stimulation

**Figure 5:**
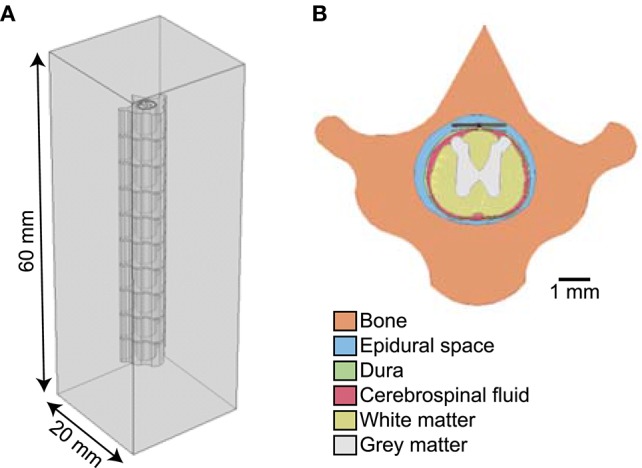
**(A)** Finite element model of rat spinal cord generated from T1-weighted MRI of thoracic level 10. **(B)** Cross section of finite element model showing the electrode placed in the epidural space above the rat spinal cord.

**Figure 6:**
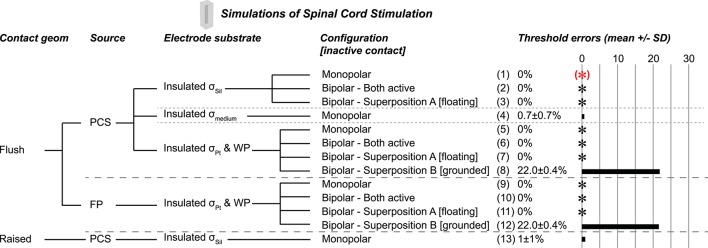
Test cases in a realistic model of rat spinal cord stimulation (SCS), all using flush contact geometry with thin platinum electrode contacts. (*)Gold standard used to compute errors in thresholds (mean ± *SD*) across nine axon location and diameter combinations. The black asterisks denote cases with zero error as compared to the gold standard. Source models: point current source (PCS), floating potential (FP). Materials: silicone (Sil), platinum (Pt), medium (muscle). WP: Insulated workplane separating contacts electrically within the electrode substrate. Superposition A: Inactive contact is floating as in the monopolar simulations. Superposition B: Inactive contact is grounded. All thresholds shown in (Supplementary Table 4).

We developed a computational model of preclinical SCS with a realistic image-based model of the rat spinal cord and overlying epidural stimulating electrode (Figure 5). The model consisted of 10 vertebrae, thoracic level 5 to lumbar level 1, created from a transverse MRI of thoracic level 10 (T10). We modeled each tissue with an appropriate electrical conductivity (Table 1) and placed the spine in a grounded box (20 mm × 20 mm × 60 mm) assigned the conductivity of muscle. In the T10 epidural space, we placed the epidural electrode: two 1.5 mm × 1 mm × 0.05 mm platinum contacts spaced 2 mm apart center-to-center along the length of the spine, backed with a 1.6 mm × 3.5 mm × 0.05 mm substrate. We simulated model axon diameters of 2, 5.7, and 10 μm at electrode-axon distances of 100, 500, and 1,000 μm. We evaluated current source implementations selected from the monopolar and bipolar cases to include implementations predicted to be either correct or inaccurate (Figure 6).

**Table 1:** Electrical conductivity of tissues and electrode components in the finite element model of spinal cord stimulation.

**Material**	**Conductivity (S/m)**	**References**
Muscle	0.2	Geddes and Baker, 1967
Bone	0.02	Kosterich et al., 1983
Epidural space^*^	0.2	Geddes and Baker, 1967
Dura	0.03	Struijk et al., 1993
Cerebrospinal fluid	1.8	Baumann et al., 1997
White matter	0.6 (longitudinal)	Ranck and Bement, 1965
	0.083 (radial)	
Gray matter	0.23	Latikka et al., 2001
Platinum	9.43^*^10^6^	Serway, 2006
Silicone	10^−12^	Davis, 1959

* *Assume that epidural space has the same conductivity as muscle*.

## Results

Our simulations using different boundary conditions to model a three-dimensional electrode used as a current source for neural stimulation revealed the following parameters to achieve accurate results:

1. ***Contact geometry***: Model the physical extent of each contact, whether raised above or flush with the surface of the substrate.
2. ***Contact model***: Model each contact as a thin domain with σ_Pt_, unless small mesh elements make the geometry assembly and/or mesh prohibitive, in which case no thin domain produces modest errors (1.5 ± 1.9%, up to 4.5%) in activation thresholds of model axons.
3. ***Source***: Use a point current source (only if placed within thin platinum domain or block of platinum) or boundary current source (specify current density), since the floating potential boundary condition requires a longer solve time and the electric potential boundary condition produces inaccurate results in certain configurations.
4. ***Electrode substrate***: Use the conductivity of silicone (σ_Sil_), although this could produce numerical instability given that the large ratio with σ_Pt_ used for the contact domains is less than the square root of machine precision (see Discussion) (Kumar, personal email communication); otherwise, use σ_Pt_ with perfectly insulating boundaries for each surface between the electrode substrate and medium, as well as within the electrode substrate to electrically isolate the contacts, although this will increase runtime.

The raw thresholds for all simulations are provided in the Supplemental Material.

### Monopolar

We evaluated the effects of different representations of the electrode contacts and the electrode substrate on activation thresholds of model axons. We identified a gold standard implementation (Figure 2, case 1), using the most realistic configuration: each contact modeled as a thin platinum domain, a point source of current at the center of the active contact, and the electrode substrate assigned σ_Sil_. We computed the percent error in activation thresholds for each of the nine combinations of model axon location and diameter for the other implementations compared to this gold standard. We used the same mesh for all monopolar simulations except cases 17–19 (raised contact geometry) and cases 26–31 (no thin electrode contact domains), as described in the Methods.

#### Electrode substrate

The silicone (σ_Sil_) behaved as a perfect insulator, and assigning zero normal current density on all boundaries (i.e., “electric insulation”) between the silicone and the medium did not affect thresholds (Figure 2, case 2). We then evaluated different conductivities and boundary conditions for the substrate for two purposes: if the electrode contacts (presented below) and the substrate had the same conductivity, this eliminated both having to mesh the small contact domains and the very small conductivity ratio between silicone and platinum, which can introduce numerical instabilities. Maintaining the substrate-medium insulating boundaries, but changing the conductivity of the electrode substrate from σ_Sil_ to σ_medium_—while maintaining thin platinum contacts—did change thresholds (Figure 2, case 3) as a result of current flow between the contacts within the electrode; this would not be an issue for a true monopolar electrode design with a single contact (rather than one active contact and one inactive contact). Thus, the contacts must be electrically isolated from each other within the electrode substrate, either by assigning a sufficiently low conductivity (such as σ_Sil_) or by using perfectly insulating dividing planes (see explanation of workplane in the Methods). Indeed, when we assigned σ_Pt_ to the insulated substrate and assigned electric insulation to the workplane within the electrode, we reproduced the gold standard thresholds (Figure 2, case 4). We also reproduced these findings with source implementations other than the point of current, as described below (cases 1–4 mirrored in cases 5–8 for boundary current source, cases 9–12 for floating potential, and cases 13–16 for electric potential), with identical results except for case 16.

#### Source

**Figure 7:**
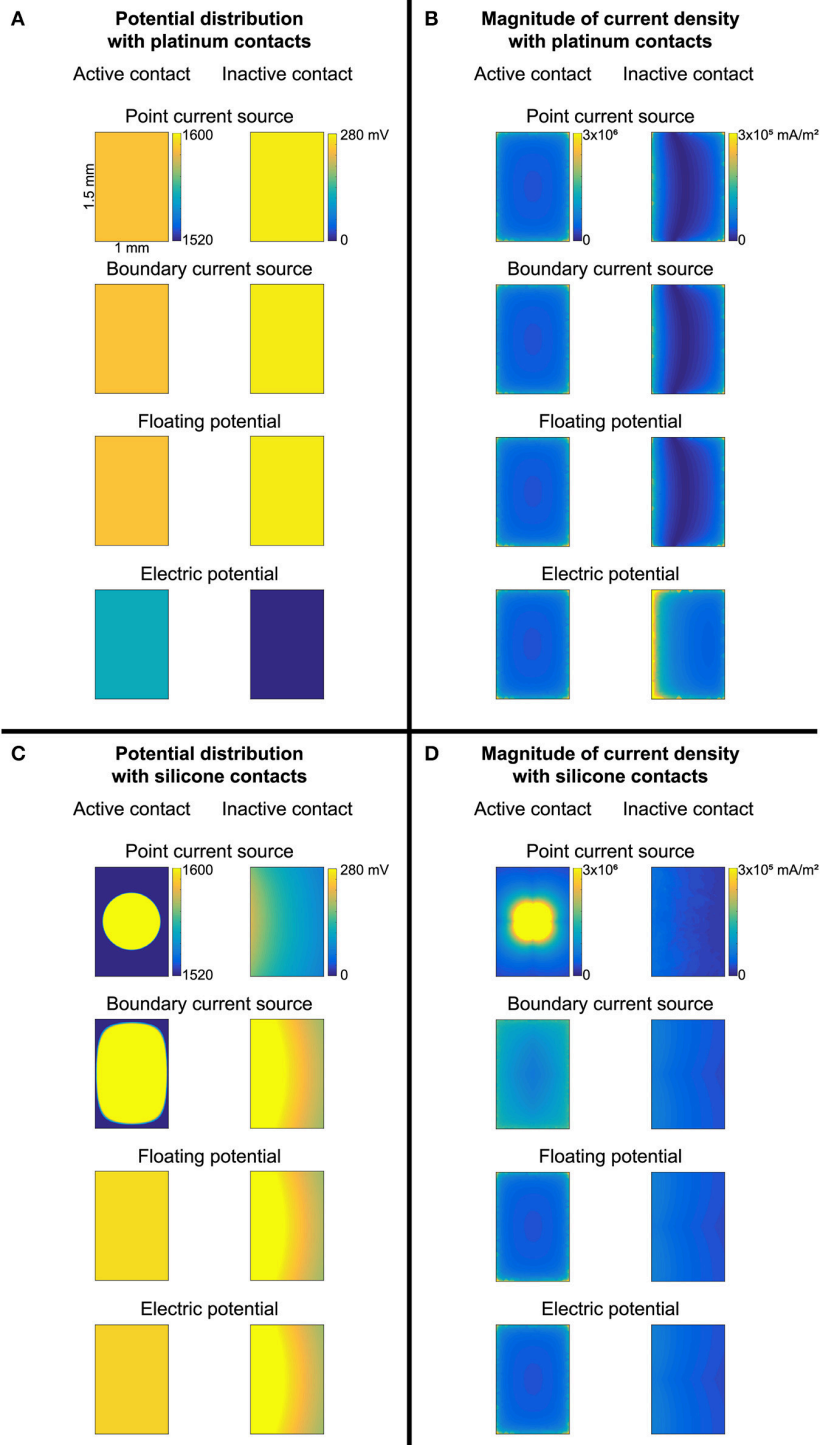
Potential distribution **(A,C)** and current density magnitude **(B,D)** over the surface of the active and inactive contacts for each of the four source models. **(A,B)** Contacts modeled as thin platinum domains embedded in a platinum substrate (with insulating boundaries between the electrode substrate and medium, as well as on the workplane within the substrate to isolate the contacts from each other). **(C,D)** Conductivity of contacts and substrate set to silicone. Note the different colorbars for the active and inactive contacts. Source models: point current source (PCS), boundary current source (BCS), floating potential (FP), electric potential (EP).

We compared the gold standard point source model to three other approaches for modeling the source, all using active boundary conditions. The source models produced identical thresholds to the gold standard when using σ_Sil_ for the electrode substrate (with or without perfectly insulated boundaries between the electrode substrate and the medium; Figure 2, zero error in cases 1–2, 5–6, 9–10, 13–14). Further, the source models produced the same thresholds after changing the conductivity of the electrode substrate to σ_medium_ (Figure 2, cases 3 vs. 7 vs. 11 vs. 15), although these thresholds were inaccurate due to current flow between the contacts (see above). However, when the entire electrode geometry was assigned σ_Pt_, and the workplane and electrode substrate-medium boundaries were set to electric insulation, three of the source implementations were accurate (Figure 2, cases 4, 8, 12), while the electric potential implementation was inaccurate (Figure 2, case 16). More specifically, with this configuration, the electric potential boundary condition on the active contact caused the inactive contact to be grounded (isopotential surface at ~0.3 μV as compared to ~0.3 V with the other three sources) (Figure 7A). This grounding of the inactive contact reshaped the potential distribution at the axon locations, thereby significantly changing the thresholds. The basis or cause for which COMSOL imposed this grounding remains unclear. This behavior would not arise for a true monopolar electrode design with a single contact (rather than one active contact and one inactive contact).

#### Contact model

A platinum domain is required for the point source of current since this source must be embedded within a conductor, but the platinum domains may be unnecessary for the active boundary sources, thereby reducing computational demands by avoiding having to mesh small domains. First, we maintained the geometry of the thin contact domains, but we assigned the same conductivity to the electrode contacts and the substrate. This resulted in 0% error with σ_Pt_, except with the electric potential source, as discussed above (Figure 2, cases 4, 8, 12, 16). When using σ_Sil_, errors arose due to the lack of isopotential on the surface of the inactive contact as it had very low rather than very high conductivity (Figure 2, cases 20, 21, 22; Figure 7A vs. Figure 7C). The errors were small with the floating potential and electric potential, but more prominent with the boundary current source, since the current density could not redistribute over the active surface. Note that if the model electrode were truly a monopole (without any other contacts, only a distant return), the issue of an isopotential inactive contact would be irrelevant. We further simplified the contact model by replacing each thin contact domain with a rectangle on the surface of the electrode substrate. The thresholds for previously accurate setups (0% error when using σ_Pt_ for the entire electrode, both contacts and substrate) yielded 1.47 ± 1.94% error (Figure 2, comparing cases 8 and 12 to cases 24 and 26) due to the changed mesh. Cases that yielded inaccurate thresholds also had slight changes in their threshold errors due to the changed mesh (Figure 2, comparing cases 20, 21, 22, 16 to cases 23, 25, 27, 28, respectively).

#### Contact geometry

Depending upon the electrode fabrication process, the electrode contact may be flush with the surface of the electrode substrate (as modeled for the results described above), raised above, or even recessed below the surface. We modified the electrode contacts in our gold standard case from flush to raised by half of the contact thickness (25 μm/2 = 12.5 μm) and found 2.4 ± 2.4% difference in excitation thresholds as compared to the gold standard for accurate implementations (Figure 2, cases 17 and 18). However, comparing the different modeling methods within the raised geometry (rather than comparing to the gold standard with flush contacts), the findings mirrored those from the flush contact cases. Specifically, thresholds were accurate when using thin platinum contacts and any of the four source models (data not shown; analogous to Figure 2, cases 1, 5, 9, 13). In addition, thresholds were accurate when using platinum for the entire electrode (contacts and substrate) with insulating boundaries between the electrode substrate and medium, as well as the insulating workplane separating contacts within the electrode substrate, except in the case of electric potentials as previously discussed (analogous to Figure 2, cases 4, 8, 12, 16). Lastly, due to the change in mesh, we again observed small errors when using an active boundary condition without meshed contacts with σ_Pt_ for the entire electrode (analogous to Figure 2, cases 24, 26, 28).

### Bipolar

When modeling an electrode with two or more contacts, superposition of the potentials generated by individually active sources is a desirable approach to solve for the potentials generated by multiple active contacts as it allows calculation of the potential distributions resulting from arbitrary combinations of contacts without re-solving the FEM.

The bipolar gold standard (Figure 3, case 1), with contacts modeled as thin platinum domains embedded in a block of silicone and a point current source centered within each contact, was analogous to the monopolar gold standard. We used the same mesh for all bipolar simulations. The test cases and corresponding threshold errors are outlined in Figure 3.

Using a silicone electrode substrate, for all four source models, grounding the inactive contact (Superposition B) produced inaccurate thresholds (~15% error) (Figure 3, cases 3, 8, 13, 18). Conversely, three of the four source models (point current source, boundary current source, and floating electrode potential) yielded accurate and identical results with simultaneously active contacts (Figure 3, cases 1, 6, 11) and with Superposition A (inactive contact floating; Figure 3, cases 2, 7, 12). While the electric potentials boundary condition produced correct thresholds with Superposition A (Figure 3, case 17), it was inaccurate with both contacts active simultaneously (Figure 3, case 16); we chose the value of the applied voltage for a given contact to deliver 1 mA when applied on its own, but with two contacts active simultaneously, the potential distribution of one contact affected the current resulting from the applied voltage at the other contact's surface.

We obtained accurate results with both contacts active or with Superposition A when we assigned σ_Pt_ to the entire electrode (contacts and substrate) rather than using platinum contacts with a silicone substrate (Figure 3, cases 4–5, 9–10, 14–15), except when using electric potentials. In this case, the inactive contact was arbitrarily grounded by COMSOL despite an applied condition of continuity (Figure 3, cases 19–20), as observed in the monopolar simulations.

### Multipolar

We used a multipolar model to determine whether the conclusions from the monopolar and bipolar simulations applied to an arbitrary number of electrode contacts. We tested two gold standard cases, either with or without non-zero current sinking to the IPG (i.e., the grounded outer boundaries of the model) (bottom panel of Figure 4). All test cases resulted in accurate thresholds, matching the appropriate gold standard (Figure 4). Specifically, we evaluated different source models (boundary current source (data not shown), point current source, and floating potential) and two electrode substrate implementations (σ_Sil_, as well as σ_Pt_ with insulating boundaries between the substrate and medium and on workplanes between the contacts). We obtained potential distributions with all contacts active and using superposition with inactive contacts floating. In all cases, we modeled the contacts as thin domains.

### Spinal cord stimulation

We evaluated different monopolar and bipolar current source implementations in a model of epidural SCS in the rat to determine whether the same implementations remained valid in an inhomogeneous anisotropic model with more complex geometry. In all cases, we modeled the contacts as thin platinum domains, and we used the same mesh for all simulations. The results were consistent with those from the simplified models (Figure 6). Specifically, the point current source and the floating potential were both valid (Figure 6, cases 1–3, 5–7, 9–11); we also obtained accurate results with the boundary current source (data not shown). Thresholds were the same with a silicone electrode substrate (Figure 6, cases 1–3 with point source) or with a platinum electrode substrate (with an insulating boundary between the electrode substrate and surrounding tissue, as well as an insulating workplane between the contacts within the electrode) (Figure 6, cases 5–7 with point source and cases 9–11 with floating potential). However, using σ_muscle_ for the electrode substrate—which demonstrates the effect of current flow between the contacts within the substrate—caused 0.7 ± 0.7% error for a monopolar source (Figure 6, case 4) vs. 3.8 ± 0.8% for the analogous simulation in the simplified model (Figure 2, case 3). The superposition results were consistent with the bipolar findings, where floating the inactive contacts (Superposition A) produced accurate thresholds, but grounding the inactive contacts (Superposition B) resulted in large errors [22.0 ± 0.4% in the SCS model (Figure 6, cases 8 and 12) vs. 14.61 ± 1.03% in the simplified model (Figure 3, cases 3, 8, 13, 18)]. Finally, when we raised the contact above the substrate (Figure 6, cases 13), the thresholds changed by 1 ± 1% (vs. 2.4 ± 2.4% in the simplified model, Figure 2, cases 17–18).

### Computational efficiency

**Figure 8:**
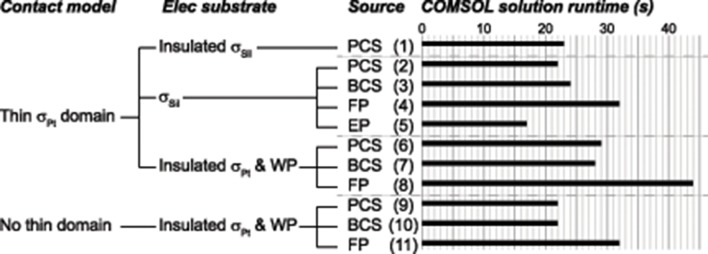
COMSOL solution runtimes for various accurate implementations. Lower numbers are desirable, indicating improved computational efficiency. The columns of labels indicate the contact model, electrode substrate model, and source model. The source models are point current source (PCS), electric potential (EP), floating potential (FP), and boundary current source (BCS). Materials: silicone (Sil), platinum (Pt). WP: Insulated workplane separating contacts electrically within the electrode substrate.

To quantify computational efficiency, we recorded solution runtimes for monopolar simulations using COMSOL's internal clock, focusing on implementations that resulted in accurate thresholds (Figure 8).

#### Electrode substrate

Using the gold standard for the source (point source) and contact model (thin platinum domain), insulating the silicone electrode substrate increased the runtime slightly (Figure 8, case 2 vs. 1). Replacing the insulated silicone with insulated platinum for the electrode substrate increased the runtime across all source models (Figure 8, comparing cases 2–4 to 6–8, respectively, for different source models).

#### Source model and contact model

Comparing source models, the floating electric potential was the slowest (Figure 8, cases 4, 8, 11), while the point current source (gold standard; Figure 8, cases 2, 6, 9) and the boundary current source (Figure 8, cases 3, 7, 10) had similar run times. These findings were consistent across electrode substrate models (Figure 8, silicone in cases 2–5 vs. insulated platinum in cases 6–8). These findings were also consistent across contact models: thin meshed platinum contact domains (Figure 8, cases 6–8) and no thin domains (Figure 8, cases 9–11), although the latter was faster for all source models given the lack of small meshed domains. The electric potential source was the fastest of the four source models (Figure 8, case 5), assuming that the model is only run once and the potential distributions are scaled *post-hoc* by the delivered current, but this source was only accurate with certain model configurations (thin platinum contacts and silicone substrate).

We observed the same trends with the rat SCS model as reported here for the monopolar simulations: we found longer solve times with added insulated boundaries and with a platinum substrate rather than silicone; from fastest to slowest, the source models were ordered EP, PCS, BCS, FP; and the solve times decreased when modeling the contacts without thin domains.

## Discussion

There are several approaches for representing current sources in finite element models (FEMs) of neuromodulation devices, and appropriate geometry, material properties, and boundary conditions are required to obtain accurate potential distributions and, subsequently, accurate activation thresholds. We evaluated different model implementations to identify approaches that resulted in accurate activation thresholds of model axons, implementations that produced small errors, and implementations that were inaccurate. We recommend using a thin platinum domain with a boundary current source for each electrode contact and a silicone electrode substrate. This approach produced accurate results and reduced runtime. We verified this approach across different electrode configurations (monopolar, bipolar, multipolar) and different model complexities (electrode in a homogeneous isotropic medium and in an anatomically accurate, inhomogeneous, anisotropic model of spinal cord stimulation). When employing superposition to solve for the potentials produced by multiple active sources, the inactive sources should be implemented with a floating boundary condition (i.e., continuity) and not grounded.

### Numerical computation software

We quantified the effects of different representations of current source stimulation of neural tissue in COMSOL Multiphysics, a commercial finite element modeling software package. We chose COMSOL given its ease of use and its widespread use in computational modeling of implanted neural stimulation devices (e.g., Gómez-Tames et al., 2014; Joucla et al., 2014; Lempka et al., 2015; Arle et al., 2016; Mourdoukoutas et al., 2017; Gunalan et al., 2018), which allows easy translation of our findings into other researchers' modeling efforts. Furthermore, COMSOL offers the option of incorporating multiple physics other than electric currents into the model in a common environment, allows user-defined differential equations, and provides easy integration with MATLAB for all steps of the model implementation and solution.

ANSYS, another commercial finite element modeling software package, is commonly used for finite element modeling, albeit infrequently for studies of neural stimulation (e.g., Brill and Tyler, 2011; Tran et al., 2015). If ANSYS is used for modeling neural stimulation, the choice of source representation should be given similar consideration. As for other numerical integration approaches, finite difference methods are not well-suited to complex geometries. Conversely, boundary element methods may be considered for neural stimulation applications given the reduced meshing requirements (only meshed boundaries, rather than meshed volumes) and capacity to solve unbounded problems (e.g., current return path at infinity). However, challenges may arise in dealing with inhomogeneous tissues, and there is a lack of commercial software packages with the range of capabilities available in FEM software packages. Further, boundary element methods are not well-suited to transient (time-dependent) or nonlinear simulations, which can both be of interest in the context of neural stimulation.

### Modeling the electrode substrate and contacts

The low conductivity of the silicone substrate prevented any current flow through the silicone either out to the tissue or between the contacts. Thus, two different approaches can be used to model the electrode substrate and contacts. First, the electrode substrate can be assigned σ_Sil_ and the contacts assigned σ_Pt_ (the gold standard). However, this could lead to numerical instabilities because the ratio of conductivities [10^−12^/(9.43^*^10^6^) ≈ 10^−19^] is less than the square root of machine precision (~10^−8^); further, given other sources of error, such as discretization, the ratio of the smallest to largest conductivities should not be less than ~10^−6^ (Kumar, personal email communication). Alternatively, the electrode substrate can be assigned σ_Pt_ with insulating boundaries between the substrate and medium, as well as on workplanes separating the contacts within the substrate. This approach requires a longer time to solve, but eliminates the small mesh elements of the electrode contact domains. Specifically, since the electrode contacts and substrate are all assigned σ_Pt_, the thin platinum domains of the contacts can be eliminated and replaced with a rectangle on the electrode's surface; either a point current source can be placed in the same location as when using thin platinum domains or the rectangle on the electrode surface can be assigned an active boundary condition. Eliminating the thin domains resulted in 1.5 ± 1.9% error in activation thresholds as compared to the gold standard, due to slight differences in the mesh. This error may be tolerable for implementation of multi-contact electrodes to simplify the model geometry and reduce the mesh demands. As discussed below, the electrode potential source did not produce accurate results with this “all platinum” approach if the electrode design had more than one contact, even if used as a monopole; rather, one of the other three source implementations must be used.

It should be noted that the conductivity of silicone varies depending upon its formulation, and various materials and fabrication processes are under investigation for novel neural stimulation electrode designs. Thus, as found in our simulations using σ_medium_ for the electrode substrate, the material properties in the FEM must be chosen to reflect accurately current flow through the substrate.

### Comparing source models

We compared four different source models: point current source, boundary current source, floating potential, and electric potential. For the monopolar simulations with platinum contacts and silicone electrode substrate, all four source models produced accurate stimulation thresholds in the simplified electrode-tissue model and realistic rat SCS model, although the solution time for the floating potential implementation was 45% longer compared to the point current source. We also tested the four source models in simulations of monopolar human deep brain stimulation (data not shown); while the point current source, boundary current source, and electric potential all produced the same activation thresholds, the floating potential did not converge due to the electrode contact's placement within anisotropic white matter. The electric potential source produced inaccurate thresholds when we changed the electrode substrate model to a slab of platinum (instead of silicone) with insulated boundaries between the electrode substrate and the medium, as well as on workplanes separating the contacts. Despite communications with COMSOL's technical support, the cause for this remains unclear. Further, while the point current source was used as the gold standard and the mesh was maintained across simulations in our evaluations, this source model requires meshing around a point, resulting in a finer mesh than for a boundary condition source model. Collectively, these findings reinforce our recommendation of the boundary current source model.

In the bipolar cases, three of the source models (point current source, boundary current source, and floating potential) produced accurate thresholds with both contacts active simultaneously or with superposition, whether using silicone for the electrode substrate or insulated platinum. The electric potential source model was inaccurate with a silicone electrode substrate with both contacts active simultaneously since the current delivered from each contact was influenced by the potential distributions in the tissue resulting from the other contact; the electric potential source was also inaccurate when using platinum for the electrode substrate, as described above for the monopolar simulations. When using superposition, we confirmed that inactive contacts must be floating, rather than grounded, so that zero net current crosses the surface of the inactive contacts. Furthermore, inactive contacts must also be approximately isopotential, which is achieved by modeling the contacts with a high conductivity; otherwise, the potential distributions at the axon locations result in errors in thresholds (0.95 ± 0.63%; Figure 2, cases 21 and 22). In the current version of COMSOL (v5.3), we tried assigning a contact impedance boundary condition to emulate platinum inactive contacts, to avoid the need to assign σ_Pt_ to a thin contact domain which increases the model runtime, but the extremely high specific resistance caused the solver to fail to converge.

It is well-established that current density is higher at the edges and corners of an electrode contact (Newman, 1966a; Wiley and Webster, 1982), as confirmed in our own source models (Figure 7). Although the boundary current source involves specifying the average current density, it nevertheless resulted in a non-uniform current density over the surface, matched to the distributions from the point current source and the floating potential.

### Verifying total current delivered

As a verification step, we integrated the current density over the outer boundaries of the model (assigned V = 0) with each source model to ensure that the expected total current was delivered. When using the electric potential source, in addition to serving as verification, we used the total current delivered in response to the 1 V source to scale the electrode potential boundary value appropriately to deliver the desired current. However, the total current was underestimated when we integrated the current density over the surface of the electrode contact rather than over the outer boundaries of the model. For example, when using a floating potential source set to 1 mA, we integrated the current density over the surface of the electrode contact using Equation 1 and obtained 0.42 mA despite the source being set to 1 mA. While the source of error is unclear despite communications with COMSOL Support, the problem might be related to integrating over platinum, given its high conductivity, in combination with insufficient mesh density for this particular calculation; refining the mesh produced a smaller error. Although there may be a conflict between integrating the current density over a surface where an active boundary condition is applied, the calculation was also inaccurate when using a point source model. While integrating the current density over the electrode surface yields an incorrect value for the total current, there are multiple accurate methods, applicable to any of the four source models, to determine the total current delivered from a single source:

1. Integrate the total current density over the outer boundaries of the model (set to V = 0) using the expression ec.normJ.
2. Same as Method 1, but using the expression ec.Jx^*^nx + ec.Jy^*^ny + ec.Jz^*^nz.
3. Integrate the reaction forces over the surface of the electrode contact using the expression reacf(V) if using an active boundary condition (FP, BCS, EP). This expression sums the reaction forces at each node on the contact boundary in response to the voltage source resulting in the current generated across the boundary.
4. Integrate the total current density over a box slightly larger than the electrode contact using the expression in Method 1 or 2 with sufficient mesh density.

Methods 1 and 2 cannot be applied to the surface of the electrode contact as the resulting total current was incorrect.

### Limitations

Although we placed the electrode in a homogeneous medium for most test cases, the results from the rat SCS model (Figure 6) and the human deep brain stimulation model (see section Comparing Source Models) matched well with those from the simplified monopolar and bipolar models (Figures 2, 3), for both accurate and inaccurate cases. Our model did not include the electrode-tissue interface, which could affect the current density distributions (Newman, 1966b; Cantrell et al., 2008). The MRG model of a mammalian axon does not include axon collaterals or terminals, but given that we sampled potentials in the neural tissue domain in COMSOL longitudinally along the axon location and transversely at different electrode-axon distances, the recommended numerical methods are expected to apply to more complex neural morphologies. Finally, since the model was purely conductive, the potential and current were linearly related. We assumed quasi-static conditions in solving the COMSOL models, thereby neglecting transients at the start and end of the current pulse, and instead approximating thresholds using the steady-state solution (Bossetti et al., 2008).

## Conclusions

Computational modeling is an important tool for analyzing and designing implanted neural stimulation devices and studying underlying mechanisms of action. We investigated different ways to represent current source electrodes, and we identified accurate and efficient implementations whether using monopolar or multipolar stimulation. We recommend modeling the contact with platinum, the electrode substrate with silicone, and either a point source of current within each contact or a boundary current source. The contact(s) and electrode substrate may be modeled using a single domain of platinum (with suitable insulating boundaries around the electrode substrate and between contacts) to remove the small contact domains if the geometry or mesh are prohibitive; this representation also avoids numerical instabilities incurred by having the range of conductivities from silicone to platinum, although the solution time is increased. Finally, when the electrode design involves more than one contact, inactive contacts must be floating (not grounded) and have a conductive surface to ensure isopotentiality. With this approach, the model need only be run once per contact and superposition can be used to compute the potential distributions resulting from an arbitrary combination of contacts each with its own current amplitude.

## Author contributions

NP and WG contributed conception of the study. NP and BT designed the study. BT performed the simulations. NP wrote the first draft of the manuscript. All authors contributed to manuscript revision, and read and approved the submitted version.

### Conflict of interest statement

The authors declare that the research was conducted in the absence of any commercial or financial relationships that could be construed as a potential conflict of interest.

## Abbreviations

BCS: boundary current source
EP: electric potential
FEM: finite element model
FP: floating potential
PCS: point current source
Pt: platinum
SCS: spinal cord stimulation
SD: standard deviation
Sil: silicone
WP: workplane.
