# Supplementary material for: Modeling Current Sources for Neural Stimulation in COMSOL
Source: Front Comput Neurosci. 2018 Jun 8;12:40. doi: 10.3389/fncom.2018.00040 (PMC6002501; doi:10.3389/fncom.2018.00040)
Supplement: Supplementary file 1 [file Table_1.docx]

Supplementary Material

Modeling Current Sources for Neural Stimulation in COMSOL

Nicole A Pelot, Brandon J Thio, Warren M Grill^*^

*** Correspondence:**Dr. Warren M Grill
warren.grill@duke.edu

# Supplementary Tables

Supp. Table 1 – Activation thresholds (mA) for monopolar simulations (Figure 2) for nine combinations of axon diameter (D) and electrode-axon distance (Dist).

| Case # | D=2 µm  Dist=50 µm | D=2 µm  Dist=750 µm | D=2 µm  Dist=2 mm | D=5.7 µm  Dist=50 µm | D=5.7 µm  Dist=750 µm | D=5.7 µm  Dist=2 mm | D=10 µm Dist=50 µm | D=10 µm  Dist=750 µm | D=10 µm  Dist=2 mm |
| --- | --- | --- | --- | --- | --- | --- | --- | --- | --- |
| 1 | 0.0539 | 0.2297 | 1.5484 | 0.0234 | 0.0727 | 0.3477 | 0.0172 | 0.0453 | 0.1711 |
| 2 | 0.0539 | 0.2297 | 1.5484 | 0.0234 | 0.0727 | 0.3477 | 0.0172 | 0.0453 | 0.1711 |
| 3 | 0.0563 | 0.2398 | 1.6117 | 0.0242 | 0.0758 | 0.3586 | 0.0180 | 0.0469 | 0.1750 |
| 4 | 0.0539 | 0.2297 | 1.5484 | 0.0234 | 0.0727 | 0.3477 | 0.0172 | 0.0453 | 0.1711 |
| 5 | 0.0539 | 0.2297 | 1.5484 | 0.0234 | 0.0727 | 0.3477 | 0.0172 | 0.0453 | 0.1711 |
| 6 | 0.0539 | 0.2297 | 1.5484 | 0.0234 | 0.0727 | 0.3477 | 0.0172 | 0.0453 | 0.1711 |
| 7 | 0.0563 | 0.2398 | 1.6117 | 0.0242 | 0.0758 | 0.3586 | 0.0180 | 0.0469 | 0.1750 |
| 8 | 0.0539 | 0.2297 | 1.5484 | 0.0234 | 0.0727 | 0.3477 | 0.0172 | 0.0453 | 0.1711 |
| 9 | 0.0539 | 0.2297 | 1.5484 | 0.0234 | 0.0727 | 0.3477 | 0.0172 | 0.0453 | 0.1711 |
| 10 | 0.0539 | 0.2297 | 1.5484 | 0.0234 | 0.0727 | 0.3477 | 0.0172 | 0.0453 | 0.1711 |
| 11 | 0.0563 | 0.2398 | 1.6117 | 0.0242 | 0.0758 | 0.3586 | 0.0180 | 0.0469 | 0.1750 |
| 12 | 0.0539 | 0.2297 | 1.5484 | 0.0234 | 0.0727 | 0.3477 | 0.0172 | 0.0453 | 0.1711 |
| 13 | 0.0539 | 0.2297 | 1.5484 | 0.0234 | 0.0727 | 0.3477 | 0.0172 | 0.0453 | 0.1711 |
| 14 | 0.0539 | 0.2297 | 1.5484 | 0.0234 | 0.0727 | 0.3477 | 0.0172 | 0.0453 | 0.1711 |
| 15 | 0.0563 | 0.2398 | 1.6117 | 0.0242 | 0.0758 | 0.3586 | 0.0180 | 0.0469 | 0.1750 |
| 16 | 0.0523 | 0.2203 | 1.5164 | 0.0211 | 0.0680 | 0.3391 | 0.0164 | 0.0445 | 0.1805 |
| 17 | 0.0563 | 0.2289 | 1.5594 | 0.0227 | 0.0719 | 0.3594 | 0.0172 | 0.0461 | 0.1836 |
| 18 | 0.0563 | 0.2289 | 1.5594 | 0.0227 | 0.0719 | 0.3594 | 0.0172 | 0.0461 | 0.1836 |
| 19 | 0.0586 | 0.2383 | 1.6250 | 0.0242 | 0.0750 | 0.3742 | 0.0180 | 0.0477 | 0.1914 |
| 20 | 0.0367 | 0.1922 | 1.4602 | 0.0172 | 0.0641 | 0.3313 | 0.0125 | 0.0406 | 0.1641 |
| 21 | 0.0547 | 0.2313 | 1.5359 | 0.0234 | 0.0719 | 0.3430 | 0.0172 | 0.0445 | 0.1688 |
| 22 | 0.0547 | 0.2313 | 1.5359 | 0.0234 | 0.0719 | 0.3430 | 0.0172 | 0.0445 | 0.1688 |
| 23 | 0.0367 | 0.1922 | 1.4711 | 0.0164 | 0.0617 | 0.3164 | 0.0125 | 0.0406 | 0.1648 |
| 24 | 0.0539 | 0.2297 | 1.5617 | 0.0227 | 0.0695 | 0.3320 | 0.0172 | 0.0453 | 0.1719 |
| 25 | 0.0547 | 0.2313 | 1.5352 | 0.0227 | 0.0695 | 0.3273 | 0.0172 | 0.0445 | 0.1688 |
| 26 | 0.0539 | 0.2297 | 1.5617 | 0.0227 | 0.0695 | 0.3320 | 0.0172 | 0.0453 | 0.1719 |
| 27 | 0.0547 | 0.2313 | 1.5477 | 0.0227 | 0.0695 | 0.3281 | 0.0172 | 0.0445 | 0.1695 |
| 28 | 0.0531 | 0.2203 | 1.5313 | 0.0219 | 0.0680 | 0.3398 | 0.0164 | 0.0445 | 0.1805 |

Supp. Table 2 – Activation thresholds (mA) for bipolar simulations (Figure 3) for nine combinations of axon diameter (D) and electrode-axon distance (Dist).

| Case # | D=2 µm  Dist=50 µm | D=2 µm  Dist=750 µm | D=2 µm  Dist=2 mm | D=5.7 µm  Dist=50 µm | D=5.7 µm  Dist=750 µm | D=5.7 µm  Dist=2 mm | D=10 µm Dist=50 µm | D=10 µm  Dist=750 µm | D=10 µm  Dist=2 mm |
| --- | --- | --- | --- | --- | --- | --- | --- | --- | --- |
| 1 | 0.0422 | 0.1836 | 1.3578 | 0.0156 | 0.0539 | 0.3188 | 0.0141 | 0.0383 | 0.1898 |
| 2 | 0.0422 | 0.1836 | 1.3578 | 0.0156 | 0.0539 | 0.3188 | 0.0141 | 0.0383 | 0.1898 |
| 3 | 0.0367 | 0.1570 | 1.1602 | 0.0133 | 0.0461 | 0.2727 | 0.0117 | 0.0328 | 0.1625 |
| 4 | 0.0422 | 0.1836 | 1.3578 | 0.0156 | 0.0539 | 0.3188 | 0.0141 | 0.0383 | 0.1898 |
| 5 | 0.0422 | 0.1836 | 1.3578 | 0.0156 | 0.0539 | 0.3188 | 0.0141 | 0.0383 | 0.1898 |
| 6 | 0.0422 | 0.1836 | 1.3578 | 0.0156 | 0.0539 | 0.3188 | 0.0141 | 0.0383 | 0.1898 |
| 7 | 0.0422 | 0.1836 | 1.3578 | 0.0156 | 0.0539 | 0.3188 | 0.0141 | 0.0383 | 0.1898 |
| 8 | 0.0367 | 0.1570 | 1.1602 | 0.0133 | 0.0461 | 0.2727 | 0.0117 | 0.0328 | 0.1625 |
| 9 | 0.0422 | 0.1836 | 1.3578 | 0.0156 | 0.0539 | 0.3188 | 0.0141 | 0.0383 | 0.1898 |
| 10 | 0.0422 | 0.1836 | 1.3578 | 0.0156 | 0.0539 | 0.3188 | 0.0141 | 0.0383 | 0.1898 |
| 11 | 0.0422 | 0.1836 | 1.3578 | 0.0156 | 0.0539 | 0.3188 | 0.0141 | 0.0383 | 0.1898 |
| 12 | 0.0422 | 0.1836 | 1.3578 | 0.0156 | 0.0539 | 0.3188 | 0.0141 | 0.0383 | 0.1898 |
| 13 | 0.0367 | 0.1570 | 1.1602 | 0.0133 | 0.0461 | 0.2727 | 0.0117 | 0.0328 | 0.1625 |
| 14 | 0.0422 | 0.1836 | 1.3578 | 0.0156 | 0.0539 | 0.3188 | 0.0141 | 0.0383 | 0.1898 |
| 15 | 0.0422 | 0.1836 | 1.3578 | 0.0156 | 0.0539 | 0.3188 | 0.0141 | 0.0383 | 0.1898 |
| 16 | 0.0352 | 0.1523 | 1.1266 | 0.0133 | 0.0445 | 0.2648 | 0.0117 | 0.0320 | 0.1578 |
| 17 | 0.0422 | 0.1836 | 1.3578 | 0.0156 | 0.0539 | 0.3188 | 0.0141 | 0.0383 | 0.1898 |
| 18 | 0.0367 | 0.1570 | 1.1602 | 0.0133 | 0.0461 | 0.2727 | 0.0117 | 0.0328 | 0.1625 |
| 19 | 0.0352 | 0.1516 | 1.1220 | 0.0133 | 0.0445 | 0.2641 | 0.0117 | 0.0320 | 0.1578 |
| 20 | 0.0352 | 0.1516 | 1.1220 | 0.0133 | 0.0445 | 0.2641 | 0.0117 | 0.0320 | 0.1578 |

Supp. Table 3 – Activation thresholds (mA) for multipolar simulations (Figure 4) for nine combinations of axon diameter (D) and electrode-axon distance (Dist).

| Case # | D=2 µm  Dist=50 µm | D=2 µm  Dist=750 µm | D=2 µm  Dist=2 mm | D=5.7 µm  Dist=50 µm | D=5.7 µm  Dist=750 µm | D=5.7 µm  Dist=2 mm | D=10 µm Dist=50 µm | D=10 µm  Dist=750 µm | D=10 µm  Dist=2 mm |
| --- | --- | --- | --- | --- | --- | --- | --- | --- | --- |
| 1 | 0.1594 | 0.4227 | 1.8438 | 0.0492 | 0.0977 | 0.275 | 0.0273 | 0.0453 | 0.1047 |
| 2 | 0.1594 | 0.4227 | 1.8438 | 0.0492 | 0.0977 | 0.275 | 0.0273 | 0.0453 | 0.1047 |
| 3 | 0.1594 | 0.4227 | 1.8438 | 0.0492 | 0.0977 | 0.275 | 0.0273 | 0.0453 | 0.1047 |
| 4 | 0.1594 | 0.4227 | 1.8438 | 0.0492 | 0.0977 | 0.275 | 0.0273 | 0.0453 | 0.1047 |
| 5 | 0.1594 | 0.4227 | 1.8438 | 0.0492 | 0.0977 | 0.275 | 0.0273 | 0.0453 | 0.1047 |
| 6 | 0.1594 | 0.4227 | 1.8438 | 0.0492 | 0.0977 | 0.275 | 0.0273 | 0.0453 | 0.1047 |

Supp. Table 4 – Activation thresholds (mA) for simulations of spinal cord stimulation (Figure 4) for nine combinations of axon diameter (D) and electrode-axon distance (Dist).

| Case # | D=2 µm  Dist=100 µm | D=2 µm  Dist=500 µm | D=2 µm  Dist=1 mm | D=5.7 µm  Dist=100 µm | D=5.7 µm  Dist=500 µm | D=5.7 µm  Dist=1 mm | D=10 µm Dist=100 µm | D=10 µm  Dist=500 µm | D=10 µm  Dist=1 mm |
| --- | --- | --- | --- | --- | --- | --- | --- | --- | --- |
| 1 | 0.2414 | 0.7875 | 1.5562 | 0.0781 | 0.1859 | 0.3203 | 0.0531 | 0.0938 | 0.1398 |
| 2 | 0.1984 | 0.6734 | 1.5078 | 0.0617 | 0.1719 | 0.3469 | 0.0445 | 0.1008 | 0.1781 |
| 3 | 0.1984 | 0.6734 | 1.5078 | 0.0617 | 0.1719 | 0.3469 | 0.0445 | 0.1008 | 0.1781 |
| 4 | 0.2469 | 0.7966 | 1.5664 | 0.0789 | 0.1867 | 0.3203 | 0.0531 | 0.0930 | 0.1398 |
| 5 | 0.2414 | 0.7875 | 1.5562 | 0.0781 | 0.1859 | 0.3203 | 0.0531 | 0.0938 | 0.1398 |
| 6 | 0.1984 | 0.6734 | 1.5078 | 0.0617 | 0.1719 | 0.3469 | 0.0445 | 0.1008 | 0.1781 |
| 7 | 0.1984 | 0.6734 | 1.5078 | 0.0617 | 0.1719 | 0.3469 | 0.0445 | 0.1008 | 0.1781 |
| 8 | 0.1625 | 0.5508 | 1.2320 | 0.0508 | 0.1406 | 0.2836 | 0.0367 | 0.0828 | 0.1461 |
| 9 | 0.2414 | 0.7875 | 1.5562 | 0.0781 | 0.1859 | 0.3203 | 0.0531 | 0.0938 | 0.1398 |
| 10 | 0.1984 | 0.6734 | 1.5078 | 0.0617 | 0.1719 | 0.3469 | 0.0445 | 0.1008 | 0.1781 |
| 11 | 0.1984 | 0.6734 | 1.5078 | 0.0617 | 0.1719 | 0.3469 | 0.0445 | 0.1008 | 0.1781 |
| 12 | 0.1625 | 0.5508 | 1.2320 | 0.0508 | 0.1406 | 0.2836 | 0.0367 | 0.0828 | 0.1461 |
| 13 | 0.2508 | 0.7938 | 1.5570 | 0.0789 | 0.1859 | 0.3188 | 0.0523 | 0.0930 | 0.1391 |
